# DT-13 Inhibits Proliferation and Metastasis of Human Prostate Cancer Cells Through Blocking PI3K/Akt Pathway

**DOI:** 10.3389/fphar.2018.01450

**Published:** 2018-12-07

**Authors:** Zhengming Wang, Yingying Wang, Shan Zhu, Yao Liu, Xin Peng, Shaolu Zhang, Zhe Zhang, Yuling Qiu, Meihua Jin, Ran Wang, Yuxu Zhong, Dexin Kong

**Affiliations:** ^1^Tianjin Key Laboratory on Technologies Enabling Development of Clinical Therapeutics and Diagnostics, School of Pharmacy, Tianjin Medical University, Tianjin, China; ^2^State Key Laboratory of Toxicology and Medical Countermeasures, Beijing Institute of Pharmacology and Toxicology, Beijing, China

**Keywords:** DT-13, prostate cancer, anti-proliferation, anti-metastasis, apoptosis, PI3K/Akt pathway

## Abstract

DT-13, a saponin monomer 13 from the dwarf lilyturf tuber, was reported to exhibit anti-inflammatory, hepatoprotective, cardioprotective as well as antitumor activities in a number of tumor cells. Prostate cancer is the second leading cause of cancer death in males, discovery of novel antitumor drug for therapy of prostate cancer is expected. Aiming to evaluate whether DT-13 could become a candidate to treat prostate cancer, we recently investigated the antitumor effect of DT-13 on human prostate cancer cells and the underlying mechanism. DT-13 was found to effectively inhibit proliferation and metastasis of prostate cancer PC3 and DU145 cell lines in a dose-dependent manner. Treatment by DT-13 resulted in a mitochondria-mediated apoptosis, which was accompanied by the chromatin condensation and nuclear shrinkage in the prostate cancer cells. Moreover, DT-13 caused remarkable upregulation of Bax, Bad, Cytochrome C, cleaved -caspase 3, -caspase 9 and -PARP, in contrast to the downregulation of Bcl-2. Nevertheless, no obvious change in intracellular ROS level was observed after DT-13 treatment. We further demonstrated that DT-13 could inhibit PC3 cell metastasis in which suppression of Integrinβ1 and MMP2/9 might be involved. Western blot analysis indicated DT-13 significantly decreased the phosphorylation of PDK1, Akt, mTOR as well as p70S6K, suggesting the pro-apoptotic and anti-metastatic effects of DT-13 on prostate cancer cells might be attributed to the blockade of PI3K/Akt pathway. Collectively, our findings suggest DT-13 is worthy of further investigation as a drug candidate for the treatment of prostate cancer.

## Introduction

Prostate cancer is the most commonly diagnosed cancer and the second leading lethal disease in males (Siegel et al., 2018). In worldwide, about 300,000 men die from prostate cancer each year (Center et al., 2012). Some types of prostate cancer grow slowly or even they would stay that way, other types are aggressive and move fast in the body. According to recent statistics, between the year of 2005 and 2011, patients with non-metastatic prostate cancer had a 98.9% five-year survival rate, but that for patients with metastatic prostate cancer is only 28.2%, suggesting most patients eventually die from cancer metastasis (Siegel et al., 2018; Wade and Kyprianou, 2018). Chemotherapeutic drugs and surgeries are common options applied in treating prostate cancer, but chemotherapy produces a series of side-effects (Perez-Gracia et al., 2018).These situations urgently require innovative pharmacotherapies to implement the control of prostate cancer development and metastasis. Recently, a number of compounds from natural products were evaluated for their chemopreventive potential against various tumors, providing new opportunity for alternative treatment of prostate cancer (Lawania and Mishra, 2013).

The steroidal saponin DT-13, [25(R,S)-ruscogenin-1-O-[β-d-glucopyranosyl-(1→2)][β-d-xylopyranosyl-(1→3)]-β-d-fucopyranoside], one of the major active compounds derived from Dwarf lillytruf tuber, has been widely studied and demonstrated to possess multiple pharmacological activities (Khan et al., 2018). This compound has shown anti-inflammatory, hepatoprotective, cardioprotective as well as immunomodulating effects with little toxicities (Khan et al., 2018). Besides, it is highlighted that DT-13 exhibited great potential in combating cancer against a variety of cancer cell lines such as lung, breast and gastric cancer, which might be attributed to its pro-apoptotic effect on cancer cells (Zhang et al., 2012; Ren-Ping et al., 2014; Li et al., 2016, 2017b; Wei et al., 2016; Yu et al., 2016; Du et al., 2018; Khan et al., 2018). It was also found that DT-13 could inhibit tissue factors and angiogenesis to suppress tumor metastasis (Zhang et al., 2012; Zhao R. et al., 2013).

Phosphatidylinositol-3 kinases (PI3Ks) are a family of phosphokinase that plays vital roles in multiple cellular processes, such as cell growth, differentiation, apoptosis, transcription and cell migration. PI3Ks transmit signals from various cytokines, growth factors and chemotherapeutic agents into intracellular messages by generating phospholipid PI(3,4,5)P3, which in turn activate protein kinase B (Akt) and other downstream effectors (Kong and Yamori, 2009). It is well known the PI 3-kinaseis often mutationally activated or over expressed in many cancers, which results in the development of cancer. Hence, many pharmaceutical researchers are actively developing inhibitors targeting PI3K and other key components in the pathway (Yap et al., 2015). At present, great efforts have been made to discover inhibitors of PI3K/Akt pathway for the treatment of cancer and a number of these inhibitors have been approved or are evaluated in clinical trials, such as Idelalisib, BEZ-235, ZSTK474, PI-103, XL-765, BYL-719, and PX-866 (Cohen, 2014; Kumar et al., 2015; Massacesi et al., 2016; Zhao et al., 2017).

In the present study, we set out to investigate the inhibitory effects of DT-13 on human hormone-refractory prostate cancer cells and to elucidate the molecular mechanisms that account for the therapeutic effect. We have demonstrated that DT-13 inhibited proliferation and induced apoptosis in the path of mitochondrion in PC3 and DU145 cells. We have also found DT-13 could inhibit PC3 cells migration and invasion at low concentrations. Moreover, PI3K/Akt signaling pathway was proved to play an important role in the anticancer effect of DT-13 on prostate cancer.

## Materials and Methods

### Reagents

DT-13 was purchased from Chengdu Push Bio-technology Co., Ltd. (Chengdu, China). MTT [3-(4,5-dimethyl-2-thiazolyl)-2,5-dipheny-2-H-tetrazoliumbromide] was obtained from Amresco (Solon, OH, United States). Adriamycin (ADR) and z-VAD-FMK were from Selleck (London, ON, Canada). Hoechst 33342 and propidium iodide (PI) were purchased from Sigma-Aldrich (St. Louis, MO, United States). Antibodies against Akt, phospho-Akt (T308), phospho-PDK1 (Ser241), phospho-mTOR (Ser2448), phospho-p70S6k (Thr389), PI3K-p110α, PI3K-p110β, PARP, phospho-p38 (Thr180/Thr182), phospho-ERK1/2 (Thr202/Thr204), phospho-JNK (Thr183/Thr185), Cytc, caspase-3, caspase-8, caspase-9,Integrinβ1, phospho-Integrinβ1 and β-actin were obtained from Cell Signaling Technology (Danvers, MA, United States). FITC Annexin-V apoptosis detection kit was obtained from BD Bioscience (San Jose, CA, United States). Mitochondrial membrane potential detection kit, mitochondrial isolation kit and 2′-7′-Dichlorodihydrofluorescein diacetate (DCFH-DA) were purchased from Beyontime Biotech (Nantong, China).

### Cell Culture

Human prostate cancer cell lines PC3 and DU145 were obtained from the cell bank of Chinese academy of sciences (Shanghai, China). All cells were cultured in RPMI 1640medium supplemented with 10% fetal bovine serum (Biological Industries, Kibbutz Beit-Haemek, Israel), 10 μg/ml streptomycin and 100 U/ml penicillin at 37°C in a humidified atmosphere containing 5% CO_2_.

### Cell Viability Assay

The effect of DT-13 on cell viability was determined by MTT assay as mentioned in our previous study (Zhou et al., 2016). In brief, PC3 and DU145 cells were seeded into 96-well plates separately at a density of 4 × 10^3^ cells/200 μl per well. Human peripheral blood mononuclear cells (PBMCs) were isolated from 15 ml of peripheral blood through density gradient centrifugation using Lymphoprep (DAKEWE, Shenzhen, China) and the suspensions of PBMCs were seeded into 96-well plate at the density of 8 × 10^3^ cells/200 μl per well. On the following day, different concentrations of DT-13 were added to each cell line. After 48 h treatment, 20 μl MTT reagent was added to each well for additional 4 h incubation. Then the supernatant was removed and 150 μl DMSO was added to dissolve the formazan crystals. The resulting absorbance at 490 nm was measured by using microplate reader iMark (Bio Rad, Hercules, CA, United States). IC50 values were calculated based on the data generated from downward sloping dose-response curve by GraphPad Prism 5 Software (GraphPad Software, San Diego, CA, United States).

### Flat Plate Colony Formation Assay

Flat plate colony formation assay was performed as we previously described, with a small modification (Chen et al., 2017). After treated with DT-13 (0, 2.5, 5, and 10 μM) for 48 h, PC3 and DU145 cells were harvested and pipetted well to become single-cell suspension. After that, 4 ml of 10% FBS RPMI-1640 containing 0.6% agarose was injected into 60-mm dishes as the bottom layer, and 1 × 10^4^ cells in 2 ml of 10% FBS RPMI-1640 and 0.3% agarose was put on the top. After incubation for 14 days, the colonies were fixed with 4% paraformaldehyde, and subsequently stained with crystal violet (0.5%) for 30 min. Colonies larger than 0.1 mm in diameter were counted using Image J software.

### Cell Cycle Distribution Analysis

Cell cycle distribution was analyzed by PI labeling after the cells were treated with DT-13 as we previously described (Wang R. et al., 2016). Briefly, PC3 and DU145 cells were seeded into 6-well plates separately at a density of 4 × 10^5^ cells/2 ml per well and treated with indicated concentrations of DT-13 (2.5, 5, and 10 μM) for 48 h. Subsequently, both floating and adherent cells were collected, washed with cold PBS, fixed in ethanol (70%), and finally suspended in 50 μg/ml of PI solution containing 0.5% Triton X-100 and 2% RNase A. Then the cells were placed in dark area for 30 min at 4°C and subjected to cell cycle analysis using BD FACS Verse flow cytometer (Becton Dickinson, Germany).

### Annexin V/PI Staining Assay

Detection of cellular apoptosis was carried out by using the FITC Annexin-V apoptosis detection assay as reported by us previously (Wang Y. et al., 2016). Briefly, PC3 and DU145 cells were seeded into 6-well plates separately (4 × 10^5^ cells/2 ml per well) and treated with DT-13 at indicated concentrations (2.5, 5, and 10 μM). After incubation, cells were trypsinized, washed with ice-cold PBS, suspended in 50 μl of 1 × binding buffer, and stained with 2.5 μl of FITC Annexin V and 2.5 μl of PI for 15 min at 4°C in the dark. After a final dilution with 200 μl of Annexin binding buffer, the samples were analyzed with BD FACS Verse flow cytometer (Becton Dickinson, Germany).

### Hoechst Nuclear Staining

Hoechst 33342 has been used as a fluorescent nuclear staining reagent to identify apoptotic cells. PC3 and DU145 cells were placed on cover slips in 6-well plates at a density 4 × 10^5^ cells/2 ml per well, followed by treatment with various concentration of DT-13 (0, 2.5, 5, and 10 μM) and adriamycin (5 μM) for 48 h. Afterward, the supernatant was removed and the cells were washed, fixed with formaldehyde, and incubated with pre-warmed cell culture medium containing Hoechst 33342 solution (1 μg/ml) for 15 min. Fluorescent images were visualized and captured by a DMI3000B fluorescent microscope (Leica, Germany).

### Assessment of Mitochondrial Membrane Potential (MMP)

The changes of MMP were estimated by using [5,5,6,6-Tetrachloro-1,1,3,3-tetraethylbenzimidazolylcarbocyanine iodide] (JC-1) probe as we previously described (Chen et al., 2017). PC3 and DU145 cells were seeded into 6-well plates at a density of 4 × 10^5^ cells/2 ml per well and incubated with 0, 2.5, 5, and 10 μM of DT-13 for 48 h. Then the cells were collected and washed by cold PBS, followed by another 15 min exposure to 2 μM of JC-1 at 37°C in darkness. The samples were suspended with 500 μl of JC-1 staining buffer and the changes of MMP were analyzed by a FACS Verse flow cytometer (Becton Dickinson, Germany).

### Measurement of Intracellular Reactive Oxygen Species (ROS) Levels

The fluorescent probe 2′-7′-Dichlorodihydrofluorescein diacetate (DCFH-DA) was utilized to measure the changes of intracellular ROS levels as described by us previously (Wang R. et al., 2016). Briefly, PC3 and DU145 cells were seeded into 6-well plates separately at a density of 4 × 10^5^ cells/ 2 ml per well, after incubation with indicated compounds for 24 h, cells were collected, washed with PBS for three times and incubated with 10 μM of DCFH-DA in serum free medium for 20 min. Rosup, a reactive oxygen activator, was used as a positive control. After washing with PBS to remove the unconjugated probe, the samples were dispersed in 500 μl of PBS and analyzed by flow cytometer (Becton Dickinson, Germany).

### Wound Healing Assay

Wound healing assay was carried out to analyze the effect of DT-13 on cell migration as we reported previously, with a little modification (Zhao W. et al., 2013). Briefly, PC3 and DU145 cells were, respectively seeded into 12-well plates at a density of 2.5 × 10^5^ cells/1 ml per well. After the cells reached 70–80% confluence, a 10 μl pipette tip was used to scratch across the center of the monolayer cells. Then the cells was washed with PBS to remove the detached cells and replenished with fresh medium contained lower concentrations of DT-13 (0, 1, 2, and 4 μM). After additional 24 h incubation, the migrated cells was monitored and imaged under the Olympus CKX41 microscope (Tokyo, Japan). Three images from three independent experiments were taken for quantitation.

### Transwell Migration Assay

Transwell migration assay was conducted by using a Transwell Boyden Chamber (Corning, Corning, NY, United States), fitted with a polycarbonate membrane with pore size of 8 μm as previously reported (Zhao W. et al., 2013). PC3 and DU145 cells were suspended in serum-free RPMI-1640 medium and plated into the upper chamber (5 × 10^5^ cells/2 ml per well), while the lower chamber was filled with RPMI-1640 supplemented with 10% FBS. The same concentrations of DT-13 were added to both upper and lower compartments. Following 24 h incubation at 37°C, the cells that had migrated through the membrane were fixed with ethanol, stained with 0.5% eosin. Cells from the top side were removed with a cotton swab. The migrated cells were examined and counted by using the Olympus CKX41 microscope (Tokyo, Japan) at high power. Three images from three independent experiments were taken for quantitation.

### Transwell Invasion Assay

Transwell invasion assay was used to examine the effect of DT-13 on invasive ability of PC3 cells as reported by us previously (Zhao W. et al., 2013). The transwell chamber was pretreated with matrigel (BD Biosciences, San Jose, MA, United States) and dried at room temperature. Other procedures are the same as for transwell migration assay, transwell invasion data were also obtained from the number of cells that had invaded across the membrane.

### Gelatin Zymography Assay

The gelatin zymography was performed to detect MMP-2 and MMP-9 levels in PC3 cells. The cells (4 × 10^5^ cells/2 ml per well) were seeded into 6-well plate, incubated with DT-13 (0, 1, 2, and 4 μM) for 24 h, and then concentrated by Amicon Ultra-4 as we reported previously (Zhao W. et al., 2013). The protein samples were separated on 10% SDS-PAGE containing 0.1% gelatin by electrophoresis. Afterward, the gel was washed with 2.5% Triton X-100 and then incubated for 24 h at 37°C in reaction buffer (50 mM Tris-HCl, pH 7.5, 10 mM CaCl_2_, 0.01% NaN_3_). Then the gel was stained with Coomassie Brilliant Blue R-250 (Merck, Darmstadt, Germany) in 10% acetic acid and 50% methanol and photographed by Bio-rad imager (Bio-rad ChemiDoc MP, United States).

### Western Blot Analysis

PC3 and DU145 cells were seeded into 6-well plates separately at a density of 4 × 10^5^ cells/2 ml per well. Cells were then treated with indicated concentrations of DT-13 and lysed in RIPA buffer for preparation of the whole cell lysate as we previously described (Wang R. et al., 2016). Equal amounts of protein were separated by 10–12% SDS-PAGE gels and then transferred electrophoretically to PVDF membranes. The blots were combined with primary antibodies (Akt, p-Akt, p-PDK1, p-mTOR, p-p70S6k, PI3K-p110α, PI3K-p110β, PARP, p-p38, p-ERK1/2, p-JNK, Cytc, caspase-3, caspase-8, caspase-9, Integrinβ1, p-integrinβ1, and β-actin) followed by incubation with horseradish peroxidase-conjugated secondary antibodies. Immunoreactive bands were detected with ECL solution and digitalized by scanning.

### Statistical Analysis

Data are presented as mean ± standard deviation (SD) of three independent experiments. Student’s *t*-test was carried out to determine significant differences among groups. The *p*-value < 0.05 was considered statistically significant. All of the statistical analysis was performed with GraphPad Prism 5 software.

## Results

### DT-13 Inhibited Prostate Cancer Cells Proliferation *in vitro*

**FIGURE 1:**
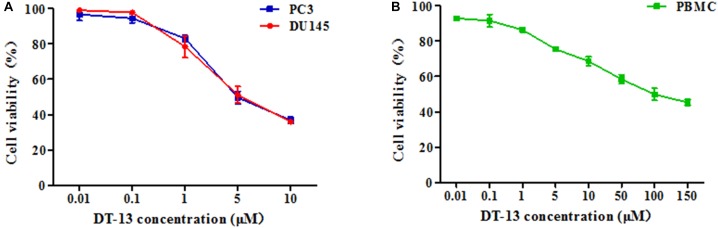
Antiproliferative activities of DT-13 on prostate cancer cells and PBMC. Antiproliferative activities of indicated concentrations of DT-13 (0.01, 0.1, 1, 5, and 10 μM) toward **(A)** PC3, DU145, and **(B)** PBMC were determined by MTT assay. Data are presented as mean ± SD, representative of three independent experiments.

**FIGURE 2:**
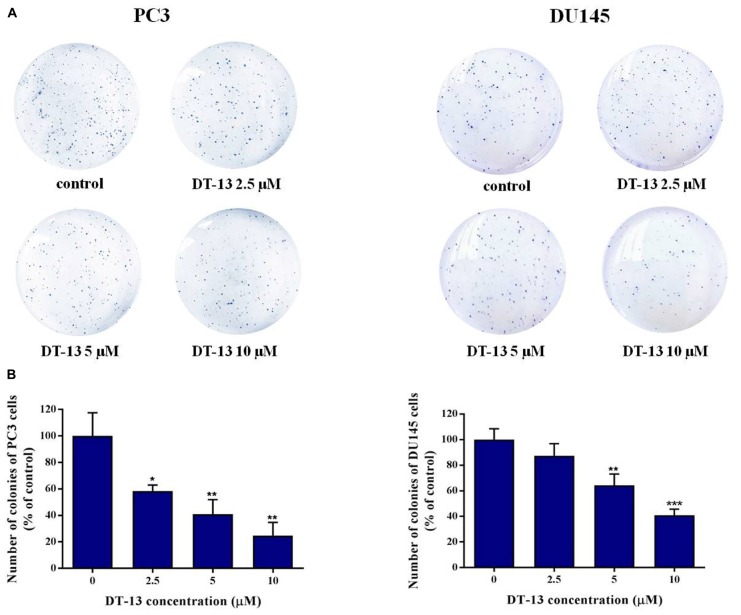
Effect of DT-13 on colony formation capability of prostate cancer cells. **(A)** Clonogenic assay was carried out to assess the effect of DT-13 on the colony formation capability of PC3 and DU145 cells. After treatment with 0, 2.5, 5, and 10 μM of DT-13 for 48 h, cells were incubated in agarose plates for 14 days and then stained with crystal violet. **(B)** The histograms represent the number of colonies of PC3 and DU145 cells following treatment with DT-13, compared to those of control cells. Data are mean ± SD (*n* = 3), representative of three independent experiments. ^∗^*P* < 0.05, ^∗∗^*P* < 0.01, ^∗∗∗^*P* < 0.001, compared with control.

To investigate the *in vitro* anticancer activity of DT-13, we examined the effect of DT-13 on the proliferation of PC3 and DU145 cell lines with MTT assay. After 48 h treatment, DT-13 inhibited PC3 and DU145 cell lines growth in a dose-dependent manner, with the IC_50_ values of 4.825 μM and 5.102 μM, respectively (Figure 1A). Besides, DT-13 showed far lower cytotoxic effect on human normal peripheral blood mononuclear cells (PBMC), with IC_50_ value of 127.8 μM (Figure 1B). Next, soft agar colony formation assay was conducted to further evaluate the tumor growth inhibitory effect of DT-13. As shown in Figure 2, both number and size of the cell colonies were decreased after DT-13 treatment, indicating that DT-13 could inhibit the colony forming abilities of PC3 and DU145 cells. Together, these results suggested DT-13 had inhibiting potential of prostate cancer cells *in vitro*.

### DT-13 Induced Apoptosis in Prostate Cancer Cells

**FIGURE 3:**
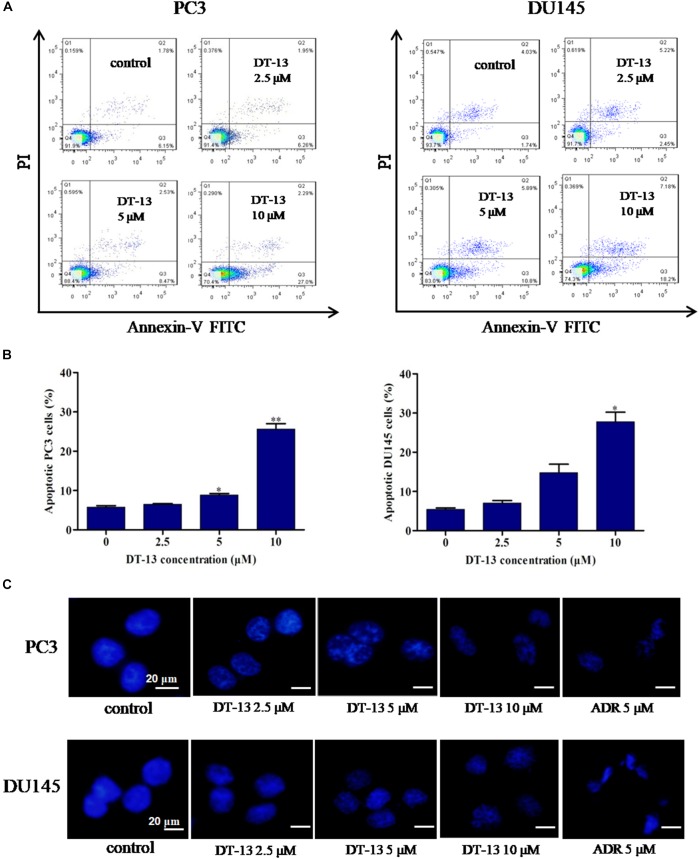
DT-13 induced apoptosis in prostate cancer cells. **(A)** PC3 and DU145 cells were treated with DT-13 at 0, 2.5, 5, and 10 μM for 48 h, stained with AnnexinV-FITC and PI, and then measured by flow cytometer. **(B)** The histograms show the percentage of apoptotic cells in PC3 and DU145 cells treated with indicated concentrations of DT-13 for 24 h. Data are mean ± SD (*n* = 3), representative of three independent experiments.^∗^*P* < 0.05, ^∗∗^*P* < 0.01, compared with control. **(C)** PC3 and DU145 cells treated with different concentrations of DT-13 or 5 μM Adriamycin (ADR) for 48 h, followed by staining with Hoechst 33342. Cytoplasmic shrinkage and nuclear fragmentation were observed under the fluorescence microscopy. Scale bar = 20 μm.

To evaluate whether DT-13 inhibited cell proliferation by inducing apoptosis in PC3 and DU145 cells, Annexin V-FITC/PI staining assay was used to measure the population of apoptotic cells. As shown in Figures 3A,B, increase of apoptotic cells was observed following DT-13 treatment. The proportions of Annexin V staining cells in 0, 2.5, 5, and 10 μM of DT-13 groups were 6.15, 6.26, 8.47, and 27.0 in PC3 cells and 1.74, 2.45, 10.8, and 18.2% in DU145 cells, indicating DT-13 induced early-phase apoptosis in both prostate cancer cell lines. More importantly, pretreatment with z-VAD-FMK, a Pan-caspase inhibitor, effectively blocked the effect of DT-13-induced apoptosis (Supplementary Figure S1A). Meanwhile, z-VAD-FMK treatment also significantly rescued cells viability after DT-13 treatment (Supplementary Figure S1B). Apoptosis is characterized by cellular shrinkage, nuclear condensation and fragmentation (Wang R. et al., 2016). Morphological assessment by Hoechst staining exhibited that chromatin condensation and nuclear shrinkage occurred in both DT-13 and ADR treated cells (Figure 3C), demonstrated the pro-apoptotic effect of DT-13 on PC3 and DU145 cells. In addition, to determine whether DT-13 can induce DNA damage, we measured the change of γH2AX, the marker for DNA double strand breaks. As shown in Supplementary Figure S2, after expose to 10 μM DT-13, the level of γH2AX had no obvious change, suggesting DT-13 couldn’t induce DNA damage in prostate cancer cells (Supplementary Figure S2). Taken together, these results indicated that DT-13 inhibited prostate cancer cells growth by inducing apoptosis.

### DT-13 Did Not Cause Obvious Change in Cell Cycle Distribution

**FIGURE 4:**
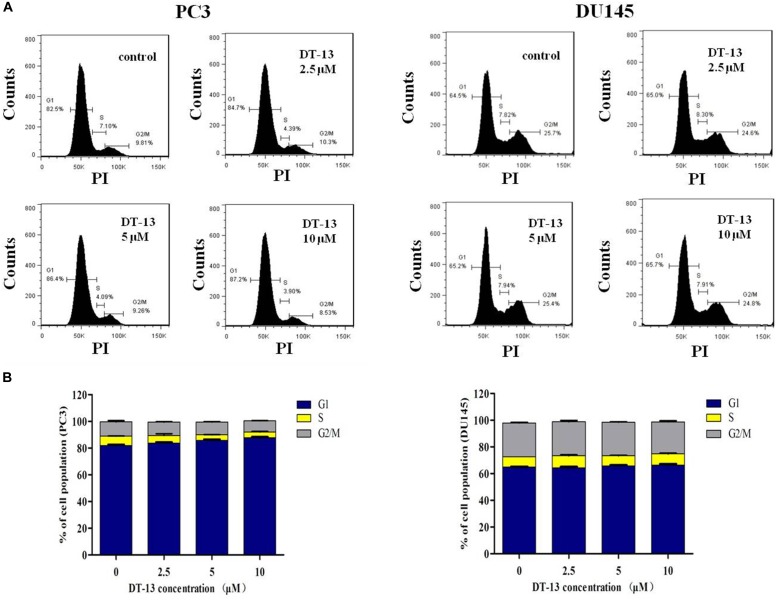
DT-13 did not affect cell cycle distribution obviously. **(A)** PC3 and DU145 cells were incubated with various concentrations of DT-13 (2.5, 5, and 10 μM) for 48 h, and the cell cycle distribution was determined by flow cytometry followed by PI staining. **(B)** Bar graphs show the percentages of PC3 and DU145 cells in G1, S, and G2/M phases. Data are presented as mean ± SD, representative of three independent experiments.

It is well established that cell cycle progress is crucial for cell proliferation, and treatment with chemical substances might cause cell senescence or apoptosis (Malumbres and Barbacid, 2009). The effect of DT-13 on cell cycle distribution was assessed by flow cytometry. DT-13 did not cause obvious change in cell cycle distribution. In PC3 cells, after treatment with 10 μM DT-13, the cell population in G1, S and G2/M phases was 87.2, 3.90, and 8.53% respectively, while that for untreated cells was 82.5, 7.1, and 9.81%. In 10 μM DT-13 treated DU145 cells, the cell population in G1, S and G2/M phases was 65.7, 4.91, and 24.8% respectively, while that for untreated cells was 64.5, 7.82, and 25.7%. The above results suggested DT-13 did not induce obvious growth arrest in PC3 and DU145 cells (Figures 4A,B).

### DT-13 Promoted Apoptosis Through Mitochondrial Pathway

**FIGURE 5:**
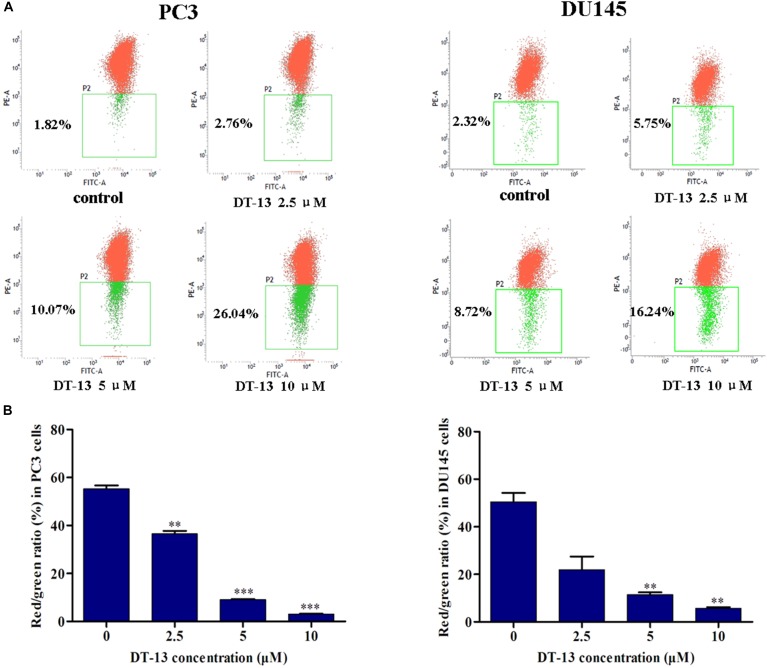
DT-13 reduced mitochondrial membrane potential (Δψm). **(A)** Cells were treated with different concentrations of DT-13 for 48 h. The Δψm was evaluated by staining with the potential sensor JC-1 and analyzed with flow cytometry. Representative dot plot (JC-1 aggregated red fluorescence and monomer green fluorescence) out of four is shown. **(B)** Statistical analysis of the relative ratio of geometric red/green fluorescence. The results represent mean ± SD of three independent experiments, ^∗∗^*P* < 0.01, ^∗∗∗^*P* < 0.001, compared with control.

**FIGURE 6:**
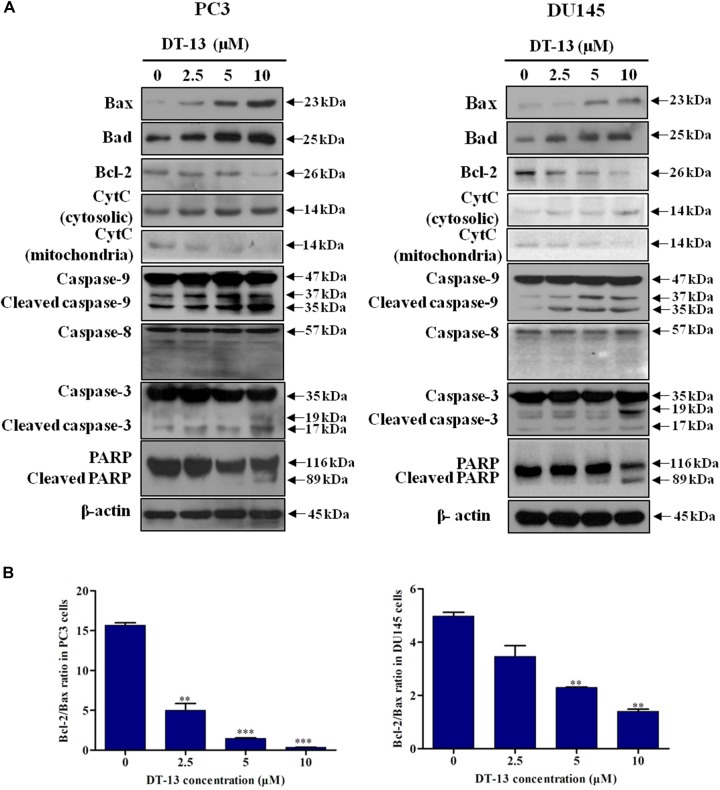
Effect of DT-13 on the expression of apoptosis-related molecules in prostate cancer cells. **(A)** Western blot analysis of the expression of cell apoptosis-related proteins after DT-13 treatment. PC3 and DU145 cells were treated with 0, 2.5, 5, and 10 μM of DT-13 for 48 h. The expression levels of Bax, Bad, Bcl-2, cytochrome c (CytC), Casapse-3, -8, -9, PARP, and cleaved Caspase-3, -8, -9, PARP were determined by western blot in both cell lines. **(B)** Bar graphs show the Bcl-2/Bax ratio in PC3 and DU145 cells. Data are mean ± SD, representative of three independent experiments (*n* = 3), ^∗∗^*P* < 0.01, ^∗∗∗^*P* < 0.001, compared with control.

Mitochondria play an important role in keeping cells alive, and interruption of mitochondrial function will trigger apoptosis. Therein, the loss of MMP is an early sign of apoptosis occurrence (Kroemer et al., 1998). We explored the changes in MMP after DT-13 treatment using the membrane permeable fluorescent probe, JC-1. As shown in Figure 5, cells in green color indicated a decrease in mitochondrial membrane potential, while those in red color indicated the higher mitochondrial membrane potential. Accordingly, red/green rate decreased in a dose-dependent way in PC3 and DU145 cells after treatment with DT-13, suggesting DT-13 could reduce mitochondrial membrane potential in prostate cancer cells. The Bcl-2 family protein was known to affect cell apoptosis by activation or inactivation of mitochondrial outer membrane permeabilization pore, which is involved in regulation of Cytochrome c (Cyto c) release into cytosol to activate caspase cascades (Slee et al., 1999; Danial, 2007). Western blot analysis demonstrated that DT-13 increased the expressions of pro-apoptotic proteins Bax and Bad, and decreased the levels of anti-apoptotic protein Bcl-2 in PC3 and DU145 cells dose-dependently (Figure 6A). And the Bcl-2/Bax radio apparently reduced after DT-13 treatment (Figure 6B). The expression level of Cyto c in mitochondria was down-regulated and that in the cytoplasm was up-regulated, suggesting DT-13 promoted Cyto c to release from mitochondria into cytosol (Figure 6A). Given that pro-caspase must undergo proteolytic activation to convert its active form to trigger apoptosis, we examined the effect of DT-13 on several key caspases and their cleaved form. As a result, the amount of cleaved caspase-9 and caspase-3 was increased in DT-13 treated cells, while that of the active caspase-8 was not detected, suggesting DT-13 might induce apoptosis through a mitochondrial-mediated intrinsic pathway. In addition, the cleavage of PARP as another sign of apoptosis was elevated after DT-13 treatment (Figure 6A). These results suggested that the apoptosis of PC3 and DU145 cells induced by DT-13 might be associated with the mitochondrial pathway.

### DT-13 Had No Obvious Effect on ROS Production

It is well known that ROS plays important roles in modulation of cell apoptosis by changing the intracellular environment (Wu, 2006). We have investigated the effect of DT-13 on ROS level and found that compared with positive control, no obvious increase of ROS was detected in DT-13 treated cells (Supplementary Figure S3), indicating that DT-13 induced mitochondrial-initiated apoptosis might not be related to oxidative stress.

### DT-13 Inhibited PC3 Cells Migration and Invasion

**FIGURE 7:**
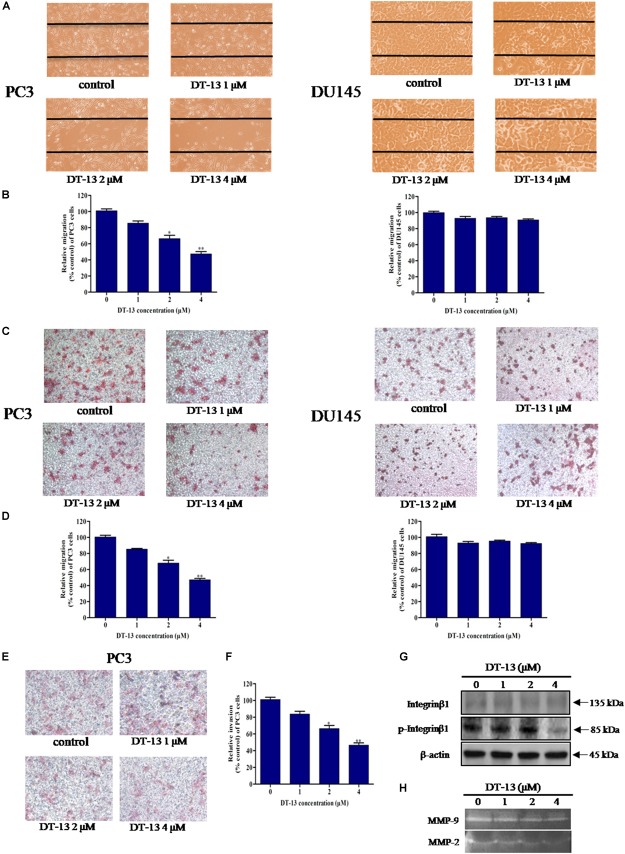
DT-13 inhibited migration and invasion of prostate cancer PC3 cells. **(A)** Migration of prostate cancer cells with or without DT-13 treatment was determined using wound healing assay. Cells migrated to the wound area were photographed and counted by inverted microcopy. **(B)** Percentages of PC3 and DU145 cells migrated to the wound area following treatment with DT-13 relative to those of the control cells. Data are mean ± SD, representative of three independent experiments (*n* = 3). ^∗^*p* < 0.05, ^∗∗^*p* < 0.01, compared with control. **(C)** Cells migration potential was assessed by Transwell migration assay. After treatment with DT-13 for 24 h, the cells migrated through the Transwell chamber membrane were measured. **(D)** Percentage of cells migrated after DT-13 treatment compared to those of control cells. Data are mean ± SD, representative of three independent experiments (*n* = 3). ^∗^*p* < 0.05, ^∗∗^*p* < 0.01, compared with control. **(E)** PC3 cells were subjected to a matrigel invasion assay with DT-13 treatment as indicated. PC3 cells invasion through the matrigel-coated chamber membrane were photographed and counted. **(F)** Percentage of PC3 cells invaded through the invasion chamber membrane after DT-13 treatment compared to those of control cells. Data are mean ± SD, representative of three independent experiments (*n* = 3). ^∗^*p* < 0.05, ^∗∗^*p* < 0.01, compared with control. **(G)** PC3 cells were exposed to the indicated concentrations of DT-13 for 24 h, then the expression level and phosphorylation level of Integrin β1 was detected by Western blot. **(H)** PC3 cells were treated with the indicated concentrations of DT-13, and the MMP2/9 activity was determined by gelatin zymography assay.

To explore the effect of DT-13 on cells migration and invasion, we performed a series of assays with DT-13 at non-cytotoxic concentrations. In wound healing assay, 1, 2 and 4 μM of DT-13 treatment for 24 h inhibited the migration of PC3 cells by 14.6, 33.5, and 52.6% respectively, compared to that for control cells, indicating that DT-13 inhibited PC3 cells migration in a dose-dependent manner (Figures 7A,B and Supplementary Figure S4). Nevertheless, DT-13 did not affect DU145 cells migration. We next used Transwell migration assay to further assess the anti-migration activity of DT-13. In accordance with the result in wound healing assay, DT-13 significantly inhibited the migration of PC3 rather than that of DU145 cells (Figures 7C,D). Subsequently, Transwell invasion assay was employed to investigate the effect of DT-13 on PC3 cells invasion capability. It was observed that following treatment with 1, 2 and 4 μM of DT-13, the number of PC3 cells that had invaded through the membrane decreased to 80.1, 70.2, and 49.3%, respectively compared to that for control cells, indicating DT-13 was capable of blocking PC3 cells invasion (Figures 7E,F). In addition, we used z-VAD-FMK to assess if the inhibition of invasion/migration is independent of cell apoptosis in PC3 cells. As shown in Supplementary Figures S5A–C, pretreatment with z-VAD-FMK did not weaken the inhibitory effect of DT-13 on cell invasion and migration. Integrinβ1 as the downstream effecter of PI3K, was reported to play a vital role in tumor migration and adhesion (Somanath et al., 2007). We have found that DT-13 inhibited the phosphorylation of Integrin β1 in a dose-dependent manner (Figure 7G). Also, z-VAD-FMK treatment couldn’t affect the reduction in phosphorylation of Integrin β1 by DT-13 (Supplementary Figure S5D). The proteolytic activities of MMP-2 and MMP-9, which reflect the ability to degrade the components of extracellular matrix, were also decreased significantly after DT-13 treatment for 24 h (Figure 7H), further proving the anti-metastatic activity of DT-13 on PC3 cells. To investigate the effect of DT-13 on EMT process in PC3 cells, we detected the protein expression of two EMT markers: Vimentin and E-cadherin in PC3 cells with or without DT-13 treatment. As expected, DT-13 treatment decreased expression of Vimentin and increased the expression of E-cadherin, suggesting DT-13 inhibited EMT process in PC3 cells (Supplementary Figure S6). Collectively, these results demonstrated that DT-13 could inhibit PC3 cell metastasis independent of cell apoptosis.

### DT-13 Stimulated Apoptosis and Inhibited Metastasis Through Blocking PI3K/Akt Signaling Pathway

**FIGURE 8:**
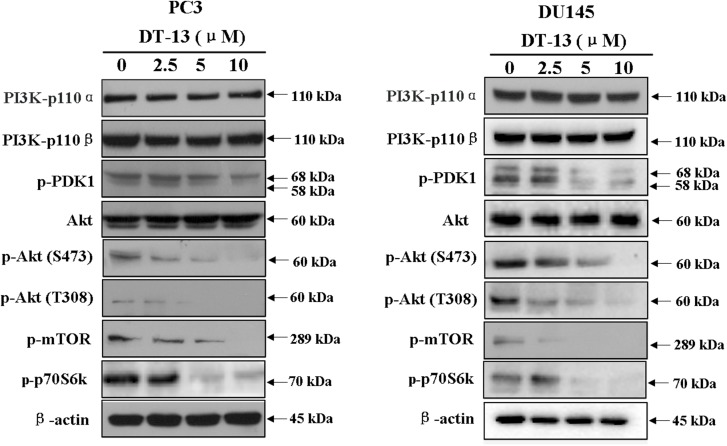
Effect of DT-13 on PI3K/Akt pathway in prostate cancer cells. PC3 and DU145 cells were treated with 0, 2.5, 5, and 10 μM of DT-13 for 48 h, then the cells were harvested, and the cell lysates were prepared to be available for western blot analysis for PI3K-p110α, PI3K-p110β, p-PDK1, p-Akt, Akt, p-mTOR and p-p70S6K.

**FIGURE 9:**
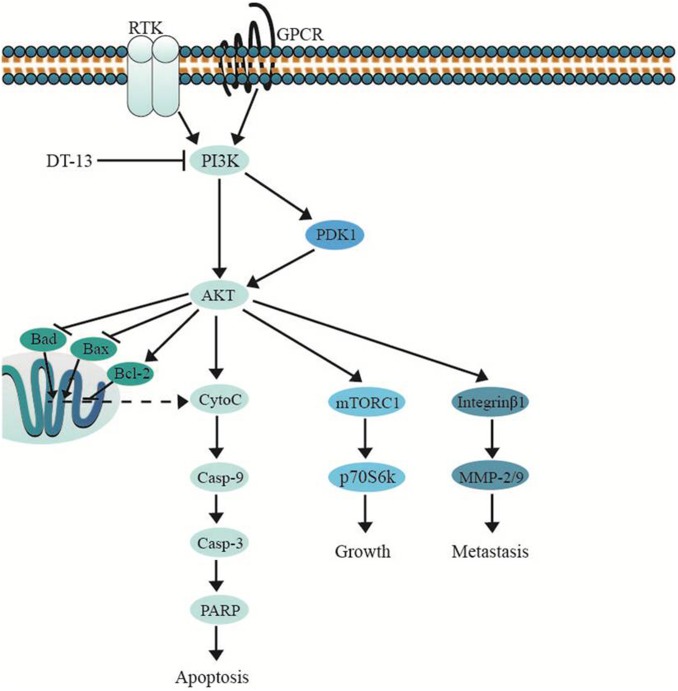
DT-13 promoted mitochondrial apoptosis and inhibited metastasis via suppressing PI3K/Akt pathway.

Several important cellular signaling pathways, such as PI3K/Akt pathway and MAPK pathway, are essential in regulating cell growth, motility, apoptosis and metastasis (Kong and Yamori, 2009; Cargnello and Roux, 2011). Therefore, we investigated whether the effects of DT-13 on PC3 and DU145 cells were related to these two pathways. Firstly, we examined the activity of DT-13 on the key signaling molecules in PI3K/Akt pathway, and the results showed that DT-13 reduced the phosphorylation of Akt, mTOR, and p70S6K in a dose-dependent manner (Figure 8). Moreover, phosphorylation of PDK1, which is the effecter of PI3K and the upstream activator of Akt was decreased. Meanwhile, there was no obvious change in the protein expression of PI3K-P110α and PI3K-P110β following DT-13 treatment. Additionally, ZSTK474, a specific PI3K inhibitor, was used as positive control to evaluate the effect of DT-13 on ROS production and ROS-activated proteins. As shown in Supplementary Figure S7A, no obvious increase of ROS was detected in either DT-13 or ZSTK474 treated prostate cancer cells. Also, the two compounds did not obviously decrease the phosphorylation of ROS activated proteins-ERK and p38 (Supplementary Figure S7B). Therefore, we can postulate that blockade of PI3K/Akt pathway might underlie the effect of DT-13 on apoptosis and migration in prostate cancer cells. There are at least three distinct MAPK signaling molecules which mediate extracellular signals into the nucleus to turn on the responsive genes in mammalian cells, including ERK, JNK and p38 kinase (Cargnello and Roux, 2011). Here, we found the phosphorylation of p38, ERK and JNK in PC3 and DU145 cells was not affected by DT-13 (Supplementary Figure S8), suggesting the anticancer effect of DT-13 was not regulated by MAPK pathway. Taken together, DT-13 promoted apoptosis and suppressed metastasis in prostate cancer cells through down-regulating the PI3K/Akt signaling axis (Figure 9).

## Discussion

Natural products have been a rich source of lead compounds in drug discovery. More than half of commercialized anticancer drugs have been identified directly from natural sources, or indirectly by structural modification based on the natural lead compound (Gordaliza, 2007). Although a lot of anticancer agents have been successfully used in the control and treatment of cancer progression, novel compounds are still needed. In this study, we have investigated the anti-cancer potential of the steroidal saponin DT-13 from Liriope muscari on prostate cancer cells. Previous study had shown the pro-apoptotic and anti-migration effect of DT-13 on lung cancer cells and gastric cancer cells. Also, Combination use of DT-13 and vinorelbine induced mitotic arrest in lung cancer NCI-H1299 cells (Zhang et al., 2012; Lin et al., 2014; Li et al., 2016, 2017a; Wei et al., 2016). However, the effect of DT-13 on prostate cancer and the underlying mechanism remain unexplored. Here, we report for the first time that DT-13 markedly inhibited proliferation and metastasis of human prostate cancer cells. We also demonstrated the potential antitumor mechanism of DT-13 involving apoptosis, migration, invasion, and PI3K/Akt pathway.

Apoptosis is an autonomic ordered programmed cell death that plays a critical role in the development and maintenance of homeostasis by eliminating damaged, aged or unnecessary cells. Impairment of this programmed cell death can result in tumorigenesis (Plati et al., 2011). Induction of apoptosis in cancer cells is known to be a key mechanism for many chemotherapeutic agents (Grutter, 2000). In this study, we observed DT-13 suppressed PC3 and DU145 cells proliferation through inducing apoptosis. To date, two major apoptotic pathways have been reported: intrinsic and extrinsic pathway (Hassan et al., 2014). Within the intrinsic apoptotic pathway, the Bcl-2 protein family plays an important role in initiating apoptosis. As one family member, Bax is considered as a pro-apoptotic protein that can form a pore in the mitochondrial membrane to allow cytochrome C release from mitochondria into the cytoplasm to activate apoptosis. The anti-apoptotic Bcl-2 prevents the release of cytochrome C, and Bad could inactivate Bcl-2 via forming a heterodimer with it, thus allowing Bax-triggered apoptosis (Giam et al., 2008). In cytoplasm, cytochrome C binds to Apaf-1 and ATP, thus activates procaspase-9, and caspase-3, -6, and -7 to trigger cell death. Activation of caspase-3 also leads to the cleavage and inactivation of PARP, which is important in keeping cell stability and survival (Slee et al., 1999; Yu et al., 2006). Our results showed after DT-13 treatment, the expression of Bax, Bad, Cyto C (cytosolic), cleaved caspase-3, -9, PARP was up-regulated, the expression of Bcl-2 and Cyto C (mitochondrial) was down-regulated, and the active caspase-8 (a sign of extrinsic pathway) was not detected, suggesting that intrinsic mitochondrial signaling pathway might be involved in DT-13-mediated cell death.

As prostate cancer cells commonly spread to lymph nodes and bones in the body, and the prognosis for patients with metastatic prostate cancer is very bad (Yu et al., 2006). So we next explored whether DT-13 had anti-metastasis activity on PC3 and DU145 cells. The results revealed that DT-13 obviously inhibited PC3 cells migration and invasion. Interestingly, DT-13 did not affect the metastasis of DU145 cells. It was reported that DU145 cells exhibits the characteristics of mesenchymal cells, and has stronger invasive potential than PC3 cells (van Bokhoven et al., 2003). We postulate that more powerful agent is required to suppress the metastasis of DU145 cells. To further explore the anti-metastasis mechanism of DT-13, we detected the effect of DT-13 on the phosphorylation of Integrin β1and the proteolytic capability of MMP-2/9. Enhanced expression of Integrin β1 was observed in prostate tumor cells, which is correlated with worse outcomes of patients (Lee et al., 2013). Downregulation of Integrin β1 could attenuate cell growth, migration, adhesion as well as angiogenesis (Howe and Addison, 2012). MMP-2/9 degrades extracellular matrix, which plays a critical role in cancer metastasis (Alaseem et al., 2017). Our result showed DT-13 could reduce the phosphorylation of Integrin β1 and inhibit the proteolytic activities of MMP-2/9, which might in turn block the migration and invasion of PC3 cells.

At last, we investigated the molecular mechanism which might correlate with the above effects of DT-13. Previously reports showed DT-13 inhibited MDA-MB-435 cell adhesion and invasion by suppressing p38MAPK, and induced apoptosis through PI3K/Akt pathway in HUVEC (Zhang et al., 2012; Qiu et al., 2014). Therefore, the effect of DT-13 on PI3K/Akt and MAPK pathways was examined in PC3 and DU145 cells. The results showed the phosphorylation of PI3K downstream effectors including PDK-1, Akt, mTOR and p70S6K was inhibited in a dose-dependent manner, while the expression of PI3K-p110αand -p110β was not changed. Akt is known to phosphorylate CREB, which could promote the expression of Bcl-2. The pro-apoptotic proteins, such as Bad and Bax, were negatively regulated by Akt through direct phosphorylation (Du and Montminy, 1998). Akt also prevents cell death by phosphorylation inactivation of Caspase-9 (Manning and Cantley, 2007). Therefore, the apoptosis induced by DT-13 might be attributed to the inhibition of Akt phosphorylation. On the aspect of cancer metastasis, it is well known that PI3K/Akt pathway plays a key role in cancer migration, invasion, and adhesion. Down-regulation of the phosphorylated Akt inhibits the proteolytic activity of MMP2/9 (Zhao W. et al., 2013). Akt phosphorylates Integrinβ1, which promotes tumor metastasis though intervention of extracellular matrix and the activity of MMPs (Somanath et al., 2007). Therefore, the anti-metastasis effect of DT-13 might be attributed to the inhibition of PI3K/Akt pathway. Moreover, DT-13 showed no effect on p38, ERK and JNK in PC3 and DU145 cells, suggesting the anticancer effect might not be related with MAPK pathway.

In summary, our findings demonstrate DT-13 can effectively induce apoptosis and inhibit metastasis in prostate cancer cells, the mechanism of which might be attributed to the blockade of PI3K/Akt signaling pathway. Since DT-13 showed weak cytotoxicity on normal cells, it is worthwhile to be further evaluated as an anticancer drug candidate for prostate cancer therapy.

## Author Contributions

ZW, YW, and SZ performed the experiments. YL, XP, and SZ analyzed the data. ZZ, YQ, and MJ prepared the figures. ZW and RW wrote the main manuscript. YZ and DK designed the experiments. All authors reviewed the manuscript.

## Conflict of Interest Statement

The authors declare that the research was conducted in the absence of any commercial or financial relationships that could be construed as a potential conflict of interest.
