# Corrigendum: DT-13 Inhibits Proliferation and Metastasis of Human Prostate Cancer Cells Through Blocking PI3K/Akt Pathway

**DOI:** 10.3389/fphar.2020.00462

**Published:** 2020-04-08

**Authors:** Zhengming Wang, Yingying Wang, Shan Zhu, Yao Liu, Xin Peng, Shaolu Zhang, Zhe Zhang, Yuling Qiu, Meihua Jin, Ran Wang, Yuxu Zhong, Dexin Kong

**Affiliations:** ^1^ Tianjin Key Laboratory on Technologies Enabling Development of Clinical Therapeutics and Diagnostics, School of Pharmacy, Tianjin Medical University, Tianjin, China; ^2^ State Key Laboratory of Toxicology and Medical Countermeasures, Beijing Institute of Pharmacology and Toxicology, Beijing, China

**Keywords:** DT-13, prostate cancer, anti-proliferation, anti-metastasis, apoptosis, PI3K/Akt pathway

In the original article, there was a mistake in **Figure 2** as published. The images that represent a minor part of the whole pictures were used in the original published paper. The corrected **Figure 2** appears below, with the new images covering over 90% of the whole pictures.

The authors apologize for this error and state that this does not change the scientific conclusions of the article in any way. The original article has been updated.

**Figure 2:**
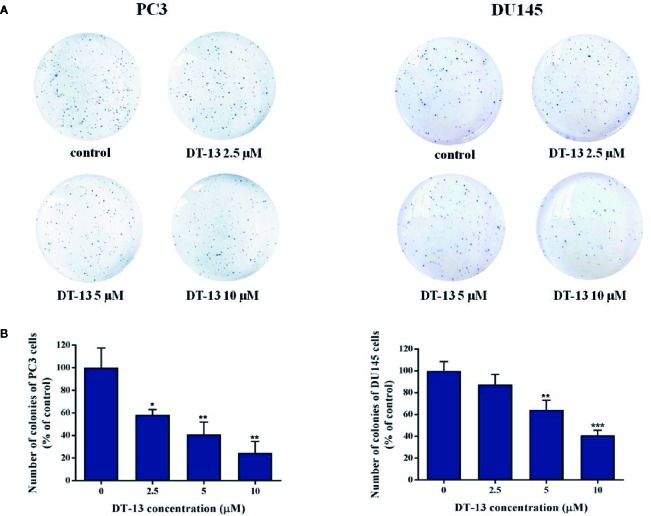
Effect of DT-13 on colony formation capability of prostate cancer cells. **(A) ** Clonogenic assay was carried out to assess the effect of DT-13 on the colony formation capability of PC3 and DU145 cells. After treatment with 0, 2.5, 5, and 10 μM of DT-13 for 48 h, cells were incubated in agarose plates for 14 days and then stained with crystal violet. **(B)** The histograms represent the number of colonies of PC3 and DU145 cells following treatment with DT-13, compared to those of control cells. Data are mean ± SD (n = 3), representative of three independent experiments. *P< 0.05, **P< 0.01, ***P< 0.001, compared with control.

