# Supplementary material for: DT-13 Inhibits Proliferation and Metastasis of Human Prostate Cancer Cells Through Blocking PI3K/Akt Pathway
Source: Front Pharmacol. 2018 Dec 7;9:1450. doi: 10.3389/fphar.2018.01450 (PMC6292965; doi:10.3389/fphar.2018.01450)
Supplement: Supplementary file 1 [file Table_1.DOCX]

Supplementary Material

DT-13 Inhibits Proliferation and Metastasis of Human Prostate Cancer Cells through Blocking PI3K/Akt Pathway

**Zhengming Wang^1^, Yingying Wang^1^, Shan Zhu^1^, Yao Liu^1^, Xin Peng^1^, Shaolu Zhang^1, 2^, Zhe Zhang^1^, Yuling Qiu^1^, Meihua Jin^1^, Ran Wang^1*^, Yuxu Zhong^2*^, Dexin Kong^1*^**

^1^Tianjin Key Laboratory on Technologies Enabling Development of Clinical Therapeutics and Diagnostics, School of Pharmacy, Tianjin Medical University, Tianjin, China

^2^State Key Laboratory of Toxicology and Medical Countermeasures, Beijing Institute of Pharmacology and Toxicology, Beijing, China

*** Correspondence:**Ran Wang
wangran@tmu.edu.cn

Yuxu Zhong

[yuxuzhong2008@aliyun.com](mailto:yuxuzhong2008@aliyun.com)

Dexin Kong

[kongdexin@tmu.edu.cn](mailto:kongdexin@tmu.edu.cn)


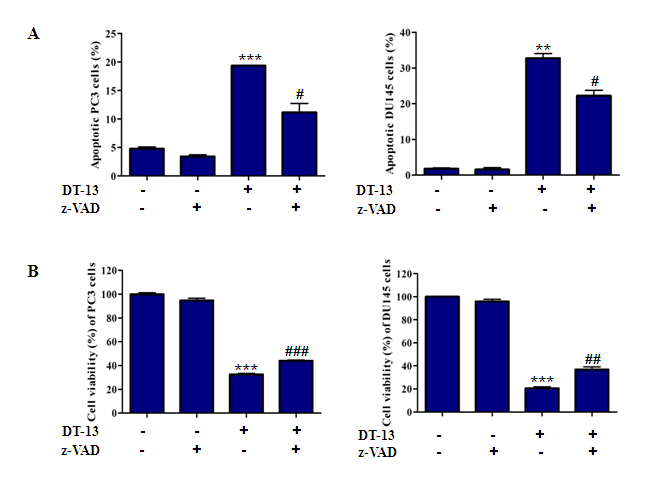


**Figure S1.** **Z-VAD-FMK blocked the effect of DT-13-induced apoptosis and rescued cells proliferation.** (A, B) PC3 and DU145 cells were treated with DT-13 (10 μM) with and without z-VAD-FMK (20 μM) respectively. (A) The percentage of apoptotic cells was determined by Annexin V/PI staining. (B) Cell viability was measured using MTT assay in PC3 and DU145 cells. For (A, B), Data are mean ± SD (n=3), representative of three independent experiments. **: P<0.01, ***: P<0.001, compared with control; ##: P<0.01, ###: P<0.001, compared with cells treated with DT-13. z-VAD: z-VAD-FMK


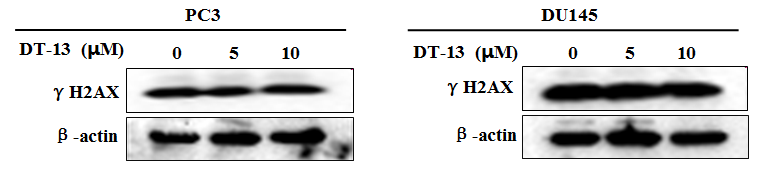


**Figure S2.** **The effect of DT-13 on the expression of γH2AX.** PC3 and DU145 cells were treated with indicated concentrations of DT-13 for 48 h, the protein levels of γH2AX were detected by Western blot.


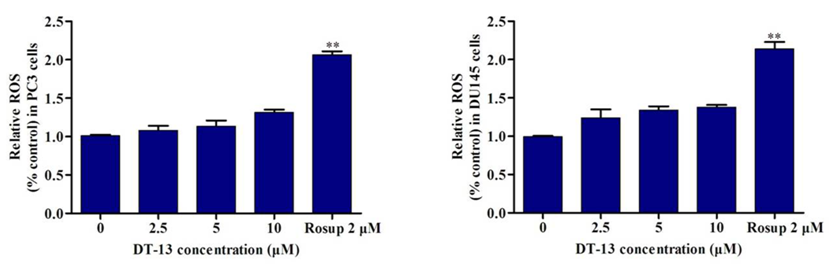


**Figure S3.** **DT-13 did not affect reactive oxygen species (ROS) production.** Cells were treated with indicated concentrations of DT-13 for 24 h, followed by DCFH-DA (10 μM) staining for 20 min. Rosup was used as a positive control to induce obvious ROS production. The ROS level was determined by flow cytometer, and expressed as fold over that in untreated cells. Data are mean ± SD, representative of three independent experiments (n = 3). **: p < 0.01, compared with control.


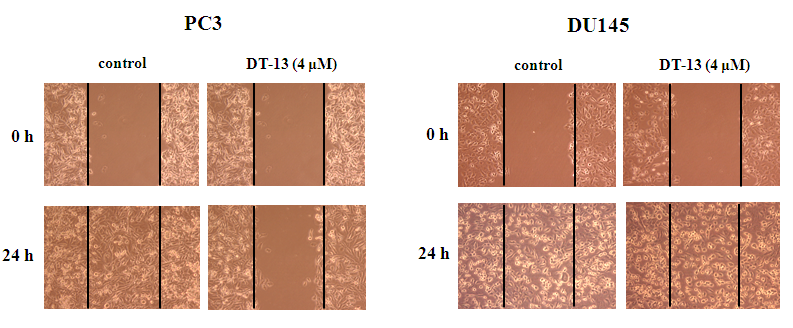


**Figure S4.** **DT-13 inhibited migration of PC3 cells.** Migration of PC3 and DU145 cells with or without DT-13 treatment was determined using wound healing assay.


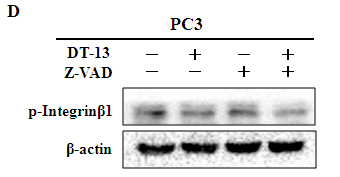

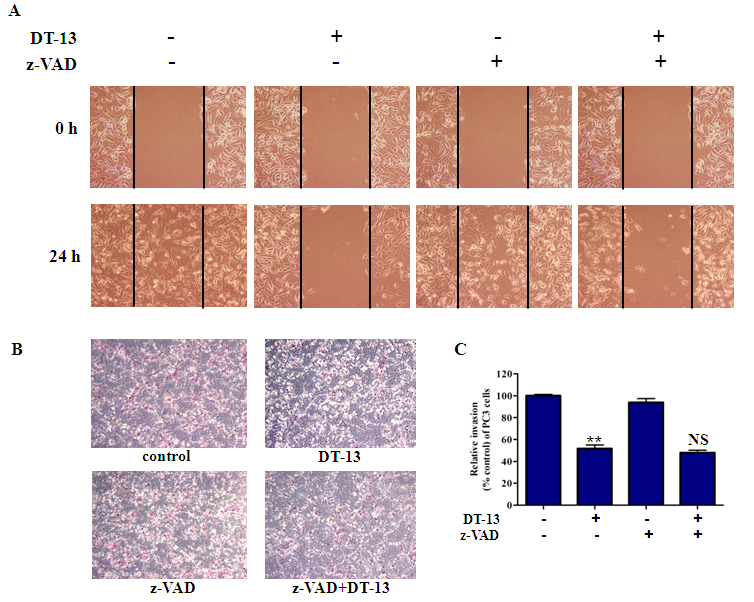


**Figure S5.** **Z-VAD-FMK didn’t reduce the effect of DT-13-inhibited PC3 cells metastasis.** (A-D) PC3 cells were treated with DT-13 (4 μM) with and without z-VAD-FMK (20 μM) respectively. (A) Cells migration potential was assessed by wound healing assay. (B) Cells invasion potential was assessed by Transwell invasion assay. (C) Percentage of PC3 cells invaded through the invasion chamber membrane. Data are mean ± SD, representative of three independent experiments (n = 3). **: p < 0.01, compared with control. NS: no significance, compared with cells treated with DT-13. (D) The phorsphorylation level of Integrin β1 was detected by western blot.


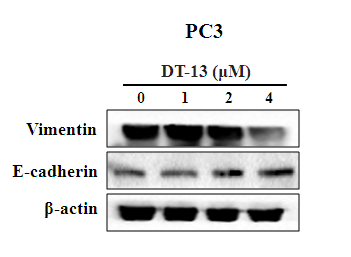


**Figure S6.** **The effect of DT-13 on the protein expression of EMT markers in PC3 cells.** PC3 cells were treated with indicated concentrations of DT-13 for 24 h, the protein level of Vimentin and E-cadherin was detected by western blot.


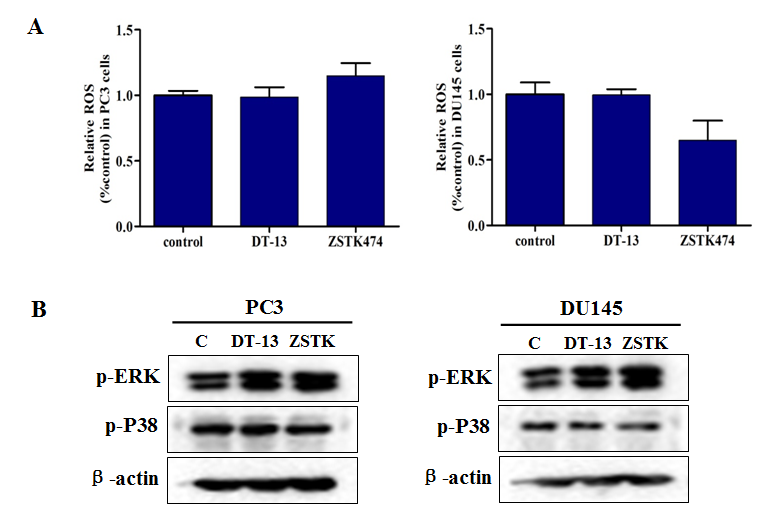


**Figure S7.** **Effects of DT-13 and PI3K inhibitor on ROS production and the phosphorylation level of relative proteins.** (A, B) PC3 and DU145 cells were treated with DT-13 (10 μM) or ZSTK474 (4 μM) for 24 h. (A) Following staining with DCFH-DA (10 μM) or 20 min, the ROS level was determined by flow cytometer. (B) The phosphorylation level of ERK and P38 was detected by western blot.


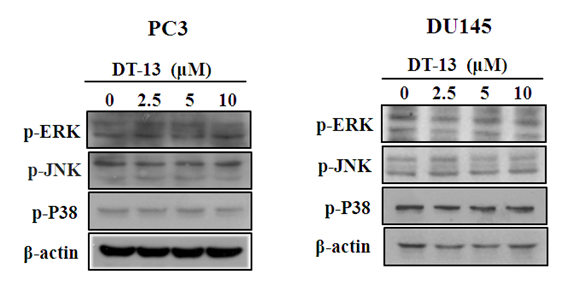


**Figure S8.** **Effect of DT-13 on MAPK pathway in prostate cancer cells.** PC3 and DU145 cells were treated with indicated concentrations of DT-13 for 48 h, and then analyzed by western blot assay for detecting p-ERK, p-JNK and p-P38.
